# Creating a data warehouse to support monitoring of NSQHS blood management standard from EMR data

**DOI:** 10.1186/s12911-024-02732-8

**Published:** 2024-11-22

**Authors:** David Cheng-Zarate, James Burns, Cathy Ngo, Agnes Haryanto, Gregory Duncan, David Taniar, Michael Wybrow

**Affiliations:** 1 Faculty of Information Technology, Monash University, Melbourne, Australia; 2 Eastern Health, Melbourne, Australia; 3 Faculty of Medicine, Nursing and Health Sciences, Monash University, Melbourne, Australia

**Keywords:** Blood management, Clinical data warehouse, Dashboard, EMR

## Abstract

**Background:**

Blood management is an important aspect of healthcare and vital for the well-being of patients. For effective blood management, it is essential to determine the quality and documentation of the processes for blood transfusions in the Electronic Medical Records (EMR) system. The EMR system stores information on most activities performed in a digital hospital. As such, it is difficult to get an overview of all data. The National Safety and Quality Health Service (NSQHS) Standards define metrics that assess the care quality of health entities such as hospitals. To produce these metrics, data needs to be analysed historically. However, data in the EMR is not designed to easily perform analytical queries of the kind which are needed to feed into clinical decision support tools. Thus, another system needs to be implemented to store and calculate the metrics for the blood management national standard.

**Methods:**

In this paper, we propose a clinical data warehouse that stores the transformed data from EMR to be able to identify that the hospital is compliant with the Australian NSQHS Standards for blood management. Firstly, the data needed was explored and evaluated. Next, a schema for the clinical data warehouse was designed for the efficient storage of EMR data. Once the schema was defined, data was extracted from the EMR to be preprocessed to fit the schema design. Finally, the data warehouse allows the data to be consumed by decision support tools.

**Results:**

We worked with Eastern Health, a major Australian health service, to implement the data warehouse that allowed us to easily query and supply data to be ingested by clinical decision support systems. Additionally, this implementation provides flexibility to recompute the metrics whenever data is updated. Finally, a dashboard was implemented to display important metrics defined by the National Safety and Quality Health Service (NSQHS) Standards on blood management.

**Conclusions:**

This study prioritises streamlined data modeling and processing, in contrast to conventional dashboard-centric approaches. It ensures data readiness for decision-making tools, offering insights to clinicians and validating hospital compliance with national standards in blood management through efficient design.

## Background

The Australian Commission on Safety and Quality in Health Care has 8 National Safety and Quality Health Service (NSQHS) standards to ensure health services provide consistent and quality care to consumers. All Australian public hospitals must demonstrate compliance to the NSQHS standards through a process of “accreditation”. This is typically performed on a 3-year cycle.

The ADaPt EH project, under which this work is conducted, funded by the Digital Health Collaborative Research Centre, seeks to streamline the assessment process with visualisation of relevant data for assessment and clinical decision making. The NSQHS “Blood Management Standard” (Standard 7) requires organisations to be able to describe, implement and monitor systems to ensure safe, appropriate and efficient use of patient’s own blood and other blood products [1]. During accreditation, hospitals are assessed against key action points from the standard.

### Blood management

Transfusion of blood products such as Red Blood Cells (RBC), Platelets (Plt), Fresh Frozen Plasma (FFP), Cryoprecipitate and Cryodepleted Plasma in hospitals is undertaken following strict conditions and protocols. In some hospitals, prescribing, administering and monitoring of patient vital signs are documented in Electronic Medical Record (EMR) systems. This facilitates accurate, real-time documentation of a patient’s medical history and provides the opportunity for data retrieval for performance monitoring and quality improvement at an organisational level.

### Blood management documentation

It is important for hospitals to document and maintain accurate records of blood products administered. This includes details such as consent to blood product administration, blood product type, quantity, clinical indication and date/time of blood product administration. With the implementation of electronic medical records (EMR) in many Australian hospitals, this information is increasing captured electronically. Though some hospitals are still using paper-based processes, electronic documentation is the future for Australian hospitals.

#### 1. Blood product order details

Blood product transfusions are medical products and procedures that require ordering by a medical practitioner. Hospitals that use EMR systems to place these orders require doctors to specify details such as blood product type, amount of blood product, clinical indication and transfusion rates.

#### 2. Pre-transfusion checks

Blood products are usually administered by nursing staff, however before transfusions are performed, specific pre-transfusion checks are required;

Consent: Informed consent, either by the patient or medical power of attorney (MPOA) is required before blood product transfusions can be administered. These are usually paper-based as an ink signature is required. Some hospitals require staff administering blood products to acknowledge citing of consent forms in their EMR systems, thus producing a record that appropriate pre-administration checks were performed.

Compatibility: Checks for blood product compatibility with intended patient (using 3 point identification check) and patient’s blood type grouping is recorded on a blood transfusion compatibility report form. This also records matching of the unique blood transfusion product identifier provided by the blood bank to specific patients. Nurses can document compatibility checking in the EMR.

#### 3. Vital sign monitoring

Monitoring of vital signs (temperature, pulse, blood pressure and respiratory rate) are required before, during and after blood product transfusions. This is to establish that it is clinically safe to administer a transfusion and to monitor for any potential adverse effects arising during or from the transfusion. Vital signs are recorded in the EMR in these extractable data source forms:

- Pulse Rate: peripheral pulse rate and heart rate.
- Respiratory Rate
- Temperature: axillary, oesophageal, oral, and tympanic.
- Blood Pressure: systolic and diastolic pressure.

Hospital guidelines on vital sign monitoring are set by the national standards. Individual hospitals then interpret these and define their own quality measures. For this standard, the Eastern Health network requires the presence of any record in each of the four categories to be present to satisfy the criteria.

**Fig. 1:**
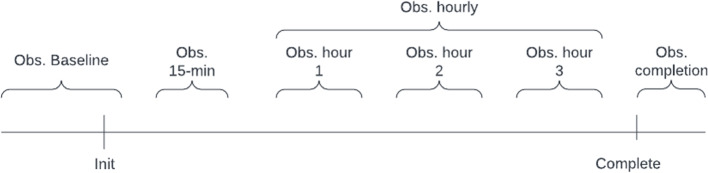
Vital Sign Observations Timeline. Estimated time intervals of each time period where at least one observation must be documented in the EMR

Similarly, the policy required for vital sign monitoring follows the specific schedule below, which is described graphically in Fig. 1:

- Baseline observations: At least one observation for each vital sign one hour prior to transfusion initiation and up to 5 mins after transfusion initiation.
- 15 Minute observations: At least one observation for each vital sign at 15 minutes after transfusion initiated with a grace period of ± 5 minutes
- Hourly observations: At least one observation for each vital sign at hourly intervals for the length of the transfusion. The first hourly observation can occur in relation to either the start of the transfusion or the 15-minute observation, with a grace period of ± 20 minutes for each hourly observation. Hourly observations are only required until the end of the transfusion, thus if the transfusion finishes before the n-th hour, then the n-th hour observation is not required.
- Post transfusion observations: At least one observation for each vital sign within 1 hour of transfusion completion. The transfusion completion time is not automated and is only recorded in the EMR when nursing staff perform a “complete transfusion” task in the EMR system.

#### 4. Transfusion completion

A transfusion can be documented in the EMR when the transfusion starts, or a clinician could decide to register it later after performing multiple transfusions and document all of them in a batch. Given that a transfusion could last for hours, the completion record would usually be registered afterwards, but it could be skipped and not be documented at all.

### EMR data

The information about the blood transfusions that were performed in a hospital is chiefly located in one single repository which is the Electronic Medical Record (EMR) system [2, 3]. It is worth mentioning that there are other hospital systems that store blood product data, but this work only focused on data recorded in the EMR due to limitations on data accessibility for other systems. The data stored in the EMR system is not only substantial, but also structured in multiple entities. This permits the EMR to contain the information independently. For example a “patients” entity or a “medical orders” entity. Thus, when a new blood transfusion in the EMR system is documented, multiple records will be inserted into these different entities. For example, one record inserted into the “medical orders” entity with the information of the blood product and another record stored into the “clinical event” entity that indicates the initiation of the blood transfusion. Consequently, the records related to a blood transfusion can be identified only by a common key attribute (e.g., the identifier of a patient’s visit) and by the timestamp of the events that occurred. This aspect will be difficult to deal with when there are consecutive or concurrent transfusions and/or specific records are not documented (e.g., lack of completion record). Blood transfusion orders in the EMR are stored in the database system as clinical events which records specific details of the transfusion order.

As a simple example, when a nurse documents a single blood transfusion, multiple clinical events are recorded as individual data points in the database (e.g. Consent data point). Each of these selected options is stored in the EMR as clinical events which are independent records in the database. Hence, the consent of a transfusion is a different record than the completion of a transfusion and so on.

### Data warehouse and analytics

An EMR system is designed to register the events that occurred in the hospital, handled as transactions. Nevertheless, this design of an operational database is not suitable to solve analytical queries. On one side, analytical queries are time-consuming and demand the resources of the operational database server. This would hinder the performance of the server and users would be affected by the slowness of the system. In addition, these heavy queries performed on the system would access unnecessary data that is not required to answer the queries, thereby making it inefficient and non-optimal. Furthermore, computation of the raw data in the operational database needs to be done at the request of the complex query every time, which is why another system needs to be created that holds specific and precomputed data to answer predefined analytical queries. A data warehouse solves the aforementioned issues that occur in operational databases since the conceptual model designed in a data warehouse models a specific subset of the data from the operational database by precomputing and aggregating the data [4].

### Related works

Multiple studies have developed Clinical Data Warehouses (CDW) focused on different topics in healthcare. However, most literature do not sufficiently describe the challenges of transforming medical data from hospital information systems.

Atay and Garani [5] developed and described the implementation of a data warehouse that aggregates lung and ovarian cancer data with the purpose of using it for data mining and decision support systems. This work does describe the database schema design of the multiple granularity levels to support particular queries efficiently due to the design but does not contain decision-based design due to the complexities of the cancer data nor the transformation process of such data. Goers et al. [6] developed and showed a custom web application that uses a CDW as a data repository focusing on the analytical results for paediatric dosing regimens. This work does show technical details and tools of the data analytics part but not on the data transformation or modelling of the CDW.

Similarly, other works mention the creation of data warehouses to support clinical decision making but they focus on the analytics performed whereas the data warehouse and data processing are not sufficiently explained. For example, Bottani et al. [7] detailed the process to train a Machine Learning model for automatic quality control of brain MRI but the development or design considerations of the CDW were not explained. Foran et al. [8] and Seneviratne et al. [9] describe dealing with the challenges of the medical data. However, the main problems in these works relate to linking multiple data sources in the CDW and the issues of handling structured and unstructured data. Kaspar et al. [10] created a CDW as a central repository of multiple information systems to automate the transformation and transfer process. However, they focus on the problems related to data linkage and description of the process and elements of the workflow.

Regarding literature where dashboards have been implemented as the end user tool for clinicians, these works mainly explain the dashboard design process rather than the CDW or other data repository that feeds the implemented dashboards. For example, Lauent et al. [11] creates a user-centred development and implementation of clinical dashboards but does not provide details of the data processing implementation. Pestana et al. [12] aimed to create dashboards that track data from a healthcare organisation to assist decision-makers. Forsman et al. [13] suggested a comprehensive integrated visualisation in the form of a patient overview for the purpose of assisting doctors in making decisions about the use of antibiotics in intensive care units. Sebaa et al. [14] created and implemented a decision support system that would help a whole region in Algeria better allocate its medical resources, describing the data warehouse schema as well as the dashboard visualisation but without detailing the data processing aspect. Weggelaar-Jansen et al. [15] developed a qualitative study focus group interviews for hospital-wide QS dashboards where the technical aspects and challenges faced were not described. Stadler et al. [16] developed, tested, and revised multiple dashboards but only using CSV files as input data for the Tableau dashboards.

Clinical data warehousing were used to support dashboards during the COVID-19 pandemic [17–19] to manage the COVID-19 outbreak and obtain insights by modelling and storing the COVID-19 and other related data, thereby focusing on the analytics and leaving behind the previous stage in the data pipeline. Other works involve other topics in healthcare areas such as radiology [20], paediatrics [6] and for other clinical decision support [21–24]. However, no past works have been published in regard to blood products for accreditation purposes and clinical decision support. Only one paper [25] that we are aware of was found which provides an overall discussion about digital accreditation dashboards, but this work gives little detail on the implementation.

Due to the multiple types of EMR records described previously, any metric calculations to be presented in a dashboard require preprocessing the extracted data from EMR since there is no single blood transfusion record that can be obtained directly from the EMR but rather it needs to be constructed from various records. Thus, this work describes the challenges and steps to process the data before calculating the performance metrics required by the national standard of blood management. Regarding data processing in past literature of Clinical Data Warehouse, most past work on CDWs or dashboards implemented in healthcare explain the usage of these tools without providing details on the challenges encountered in implementing these, they rather focus on the usage and analytics done from these tools once these are built. We only found one article regarding data processing relevant to this work which was from Atay and Garani [5] which created a cancer data warehouse but it only required straightforward preprocessing operations such as filtering, cleaning, and grouping and only a few performance metrics. Table 1 summarises the most recent Clinical Data Warehouses past works and closely relevant to our work that describes the technical aspects of Data Modelling (DM), Data Processing (DP), System Architecture (SA) and Dashboard (D).

**Table 1:** Related work summary table

Article	Goal	DM	DP	SA	D
Atay and Garani [5]	Develop CDW for cancer diseases using snowflake schema for efficient analysis	$\checkmark$	$\checkmark$	$\checkmark$	-
Goers et al. [6]	Create Pharmacokinetics CDW with genetic polymorphism analysis in pediatric patients	-	-	$\checkmark$	$\checkmark$
Bottani et al. [7]	Develop accurate ML model for automatic quality control of brain MRI scans in a CDW	-	-	$\checkmark$	-
Foran et al. [8]	Outline strategy to develop CDW for personalized treatment in precision medicine programs	-	-	$\checkmark$	-
Kaspar et al. [10]	Explore feasibility of transferring clinical routine data from CDW to an EDC	-	$\checkmark$	-	-
Agapito et al. [17]	Develop CDW integrating COVID-19 and climate data to analyze virus spread	-	-	$\checkmark$	$\checkmark$
Fleuren et al. [18]	Explain process to develop CDW of critically ill COVID patients for clinical questions	-	-	-	$\checkmark$

### Contribution

This paper makes a substantial contribution to healthcare informatics by deviating from the prevailing focus on dashboard implementation, instead underscoring the streamlined creation of a clinical data warehouse tailored for blood management. In contrast to existing literature, our work distinctly addresses the intricacies associated with data from blood transfusions extracted from EMRs, recognising the unique challenges inherent in managing and interpreting this critical healthcare information. In the “Data Extraction” section, we discuss the challenges linked to using raw data from electronic medical records (EMR) for blood transfusions. We outline the complexities of the long-format raw data which requires preprocessing to obtain the actual blood transfusions performed in the healthcare institution. We introduce a custom data model capable of storing the preprocessed wide-format data but that still requires postprocessing in the data warehouse to finally reconstruct blood transfusions. We describe the processing techniques to transform the data into the specified data model and address the calculation of performance metrics for effective blood management practices explained in the “Blood Management Documentation” subsections which can then be utilised by existing commercial visualisation systems such as Power BI and Tableau. By introducing a clinical data warehouse and associated dashboard, our work provides a resource for healthcare accreditation and decision-making at Eastern Health and similar health providers. Clinicians can leverage the visual tools embedded in the dashboard to enhance care outcomes, efficiently documenting and analysing trends in blood transfusions across different hospital branches and clinic wards over varying time periods.

The rest of this paper is organised as follows. “Methods” section describes the methods applied to process the data and build the clinical data warehouse. “Results” section presents the results obtained from this work. Finally, “Conclusions” section provides our conclusions and possible future work.

## Methods

All methods employed in this work adhere to the pertinent guidelines and regulations. It is important to mention that dummy data, representing daily extracted data from EMR, was employed and depicted in the figures to safeguard the privacy of the original data.

**Fig. 2:**
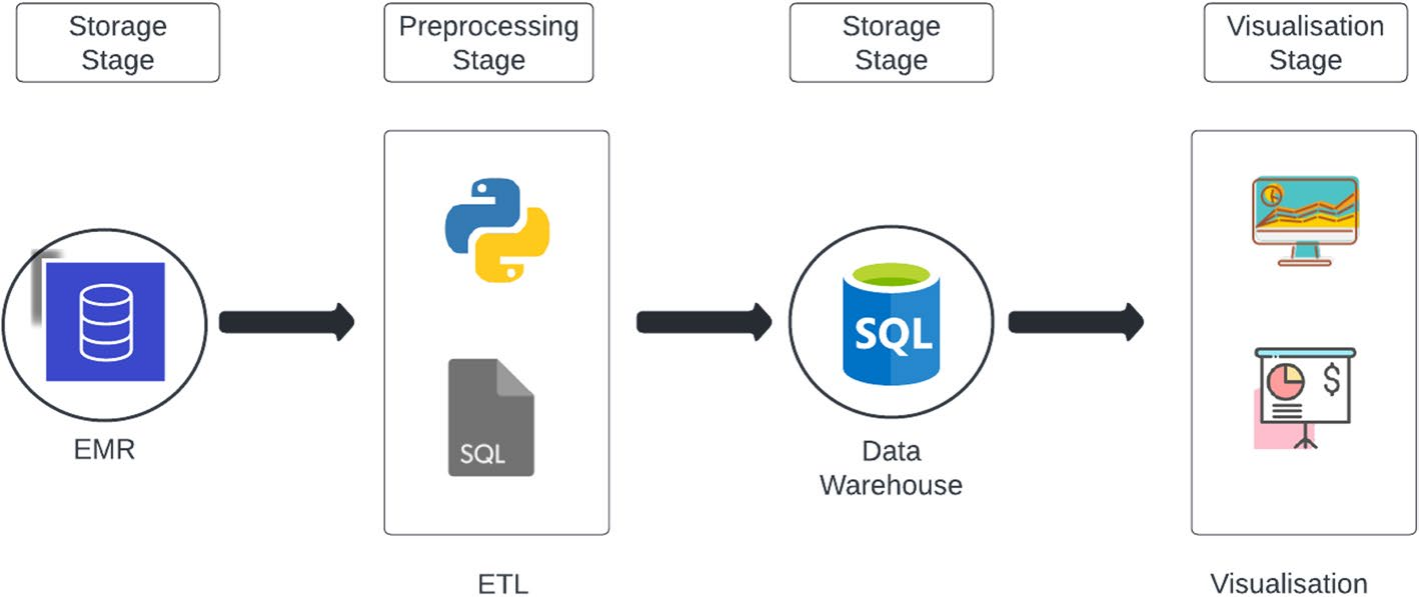
Architecture. Each stage of the data pipeline that EMR data must go through

### Architecture

The data from the EMR must go through a series of steps before it can be useful for clinicians. Figure 2 shows the overall architecture. The circular nodes (storage stages) represent the stages where the data is physically stored in a given system. The rectangular blocks represent the stages where data is processed and visualised respectively.

The first storage stage is where all the raw data is located and serves as the input of the next stage which is the Extract, Transform, Load (ETL) process. This stage refers to the operational database where all transactions that occur (e.g., blood transfusion) in the hospital system are recorded. The main objective in this stage is to explore the database and its entities that contain information about blood products. For this stage, staff from the hospital are required that possess clinical and SQL knowledge to be able to explore the data from the EMR and define the tables and which columns need to be extracted.

The ETL process consists of three steps. First the extraction of the data from any source or multiple sources. Second, the extracted data is transformed and then loaded into the data warehouse. The extraction step for this work was done using Cerner Command Language (CCL) queries given the EMR system used by our hospital partner is the Cerner Millenium database. CCL is highly similar to the most popular declarative language in database management systems, Structured Query Language (SQL). Data quality checks are primarily conducted during the extraction phase, where missing values in critical columns are filtered out in the CCL query before data extracts are generated. The correctness of data are resolved during the data validation process which happens during the development stage. Since the standard evaluates the documentation of blood management procedures, incomplete or missing data are considered part of the metrics to assess rather than issues to be resolved during processing.

To implement the processing stage in a different EMR system, only the extraction step needs to be modified to fit the extraction process according to the non-Cerner system. It is important to clarify that the remaining stages would still be valid once the columns that are used in the process are mapped. Just a column renaming would be enough to satisfy the extraction process given that the next stage expects specific column names. The transformation step is performed by a program implemented in Python to preprocess the raw data extracted from the EMR. Finally, the loading step refers to the process of inserting the transformed data, produced in the previous step, into the data warehouse using SQL statements. These SQL statements are previously defined in the Python program which has the column names and insert statements. Now, these SQL statements needed to reconstruct the blood transfusions are generated in the Python programming language given that these statements depend on the column names extracted. In case any column name is renamed, it can be easily modified in the configuration files of the Python program and the SQL statements recreated. Once these SQL statements, triggers and procedures are generated, they are pushed into the data warehouse, thereby leveraging the database engine to reconstruct the transfusions. For example, a trigger was created to find the closest “Initiate” record that has been previously inserted. This piece of code is only executed when a “Complete” record is inserted in the data warehouse. Since an “Initiate” record always occurs before a “Complete” record, a match will be found.

The third stage (i.e. second storage stage) is where the processed data is stored and where the full reconstruction of the blood transfusion is done as explained before. This stage becomes a new source of data that is designed specifically to allow efficient querying of blood product documentation.

Once the batch of data extracted from EMR is processed and now resides in the clinical data warehouse, it can be finally consumed in stage four by a visualisation system (e.g., Tableau). In this stage, the EMR data finally becomes understandable for a clinician, accreditation entity or any other person of interest in blood management.

### Data extraction

In order to extract only the necessary data from the EMR to evaluate the correct documentation for blood management, domain experts from the hospital were consulted to determine which entities in the database schema from EMR were needed. From the Oracle Cerner system, three entities were used: Patient, Encounter and Event. Thus, the CCL queries developed to extract the data from EMR were carefully designed to avoid putting a heavy workload on the operational medical database given that this could undermine this safety-critical system. The data extracted showed multiple challenges described as follows.

#### Transfusion split into multiple records

**Fig. 3:**
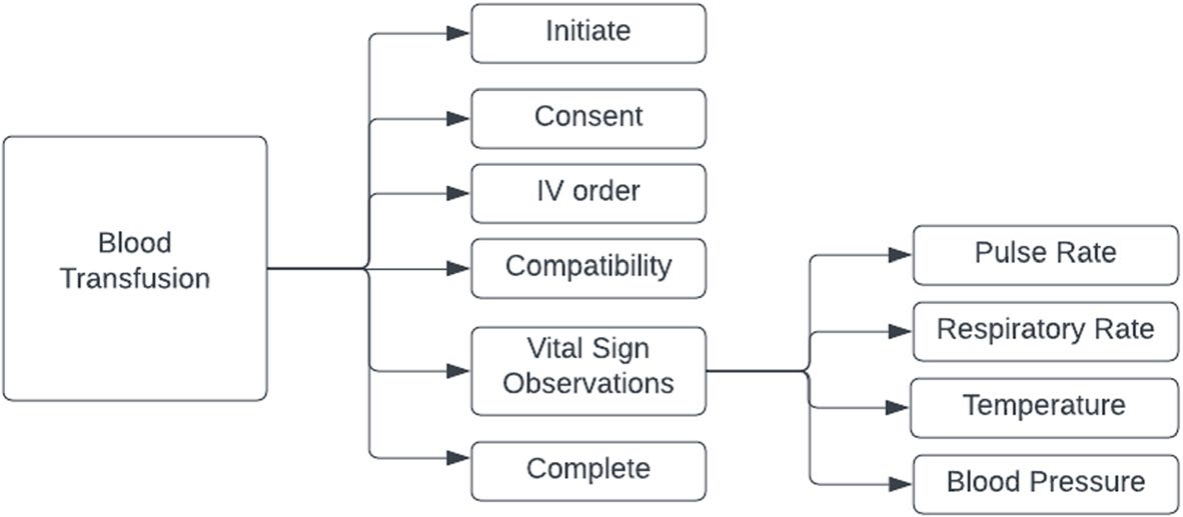
Transfusion records structure. A blood transfusion is comprised of multiple records, as are observations

Blood transfusions need to be recorded for every instance and each blood transfusion has associated medical information. Another important measure associated with a transfusion is to keep track of multiple vital signs of a patient in multiple time periods, i.e., before a transfusion starts, when it starts, during the transfusion, and at completion. Four vital signs are monitored at the aforementioned time periods to indicate the health status when a transfusion takes place. All these values can be found in the EMR as clinical events with different values for certain columns. Thus, every record involved in a blood transfusion has the same structure since they are all extracted as clinical events that are stored and performed at a specific date and time. Reconstruction of a structured version of each blood transfusion allows complex analytical queries to be performed more efficiently. Figure 3 shows the breakdown of records stored in the EMR associated with a single blood transfusion.

#### Start and end times of transfusion not linked directly

A difficult problem found in the blood transfusion extract is determining when a transfusion was completed. First, a completion record could be missing for a transfusion. This means that for some transfusions, we do not know for sure when the transfusion was completed.

Second, when a “Complete” record is found, we can only determine that the record belongs to a specific patient during a particular day. In this case, we can only assume that the “Complete” record should match the latest “Initiate” record. However, this becomes a problem when a patient has received multiple simultaneous transfusions.

#### Transfusion event records become invalid

Much data recorded in the EMR is dependent on data entry by clinical staff, thus prone to human error. There are processes in place for EMR uses to retrospectively correct EMR data when identified, for instance, correcting the start time of a procedure that happened at a different time than what was initially recorded.

**Fig. 4:**
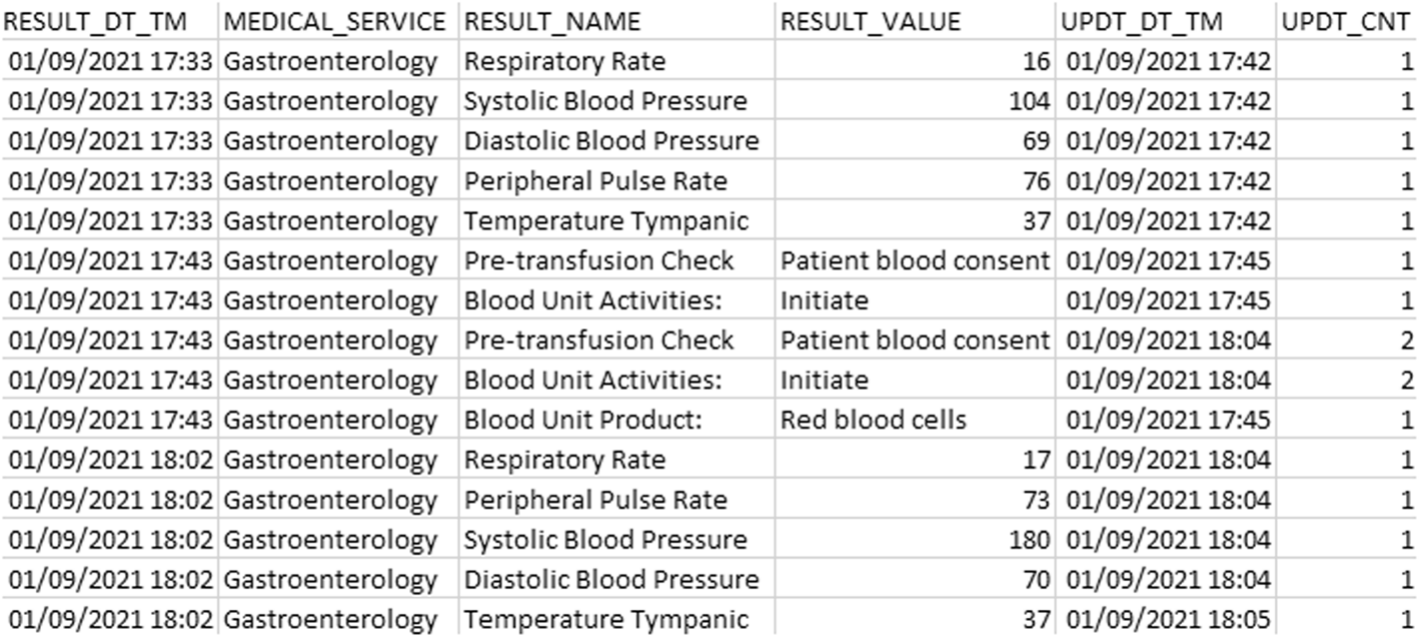
Data sample with transfusion events. Example of data extracted from EMR that contains required columns having identifier columns removed. Extract shows invalidated records due to greater values in UPDT_CNT column as well as vital sign observations before and after an “Initiate” record

The system interface provides a simple way to update the value and it just looks like a value replacement in the system. However, internally in the EMR database, an additional record is generated and the previous record with the incorrect value is kept as well, ensuring the provenance of all changes in the EMR. This additional record contains the same information except for at least three values: the value that was corrected and modified, a numerical value that contains the version of the event, and a newly generated unique record identifier. Figure 4 shows an example of this issue. Because of how data is stored in the EMR database, additional preprocessing must be done to the data once it is extracted and before inserting it in the clinical data warehouse. This process will be briefly explained later in this section.

### Performance metrics

The NSQHS Standard metrics in blood management documentation require counting the outcomes of all transfusions conducted within a specified timeframe and/or ward location, identifying how many conform or deviate from a specific metric. Consequently, prior to metric calculation, it is necessary to reconstruct each individual transfusion that took place in the hospital using raw extracted data. Figure 3 illustrates that each leaf denotes a record in the extracted dataset. Only the Initiate record signifies the commencement of a transfusion, and the subsequent records must be correlated with the initiation record. The accurate documentation of vital signs at various intervals during the blood transfusion process is crucial within the blood management standard. Figure 1 outlines the four principal intervals integral to the standard metrics. To compute the metrics detailed in the “Blood Management Documentation” section, it is necessary to associate Initiate records with each record type, including the four vital sign records, throughout the transfusion duration. Reconstructing transfusions facilitates the acquisition of numerical values for metrics, especially those related to vital sign monitoring. This involves not only matching the four vital sign records across the four intervals but also disaggregating hourly observations into three subintervals, depending on the presence of a completion record before the maximum three hours. The intricacies of assembling the raw data, as explained in the previous section, and the NSQHS Standard metrics requiring the reconstruction of transfusions, coupled with the potential invalidation of records in subsequent data extractions, contribute to the heightened complexity of metric calculations compared to other studies where healthcare analytics dashboards or data warehouses are implemented.

### Data preprocessing

Preprocessing of data is a vital step to ensure the validity and correctness of the entire process. Given the complexity of the raw data retrieved from EMR described previously, data must be prepared and processed to obtain an accurate clinical data warehouse. Next, we explain the steps to deal with each of the data challenges explained in the Data Extraction section.

#### Transfusion split into multiple records

**Fig. 5:**
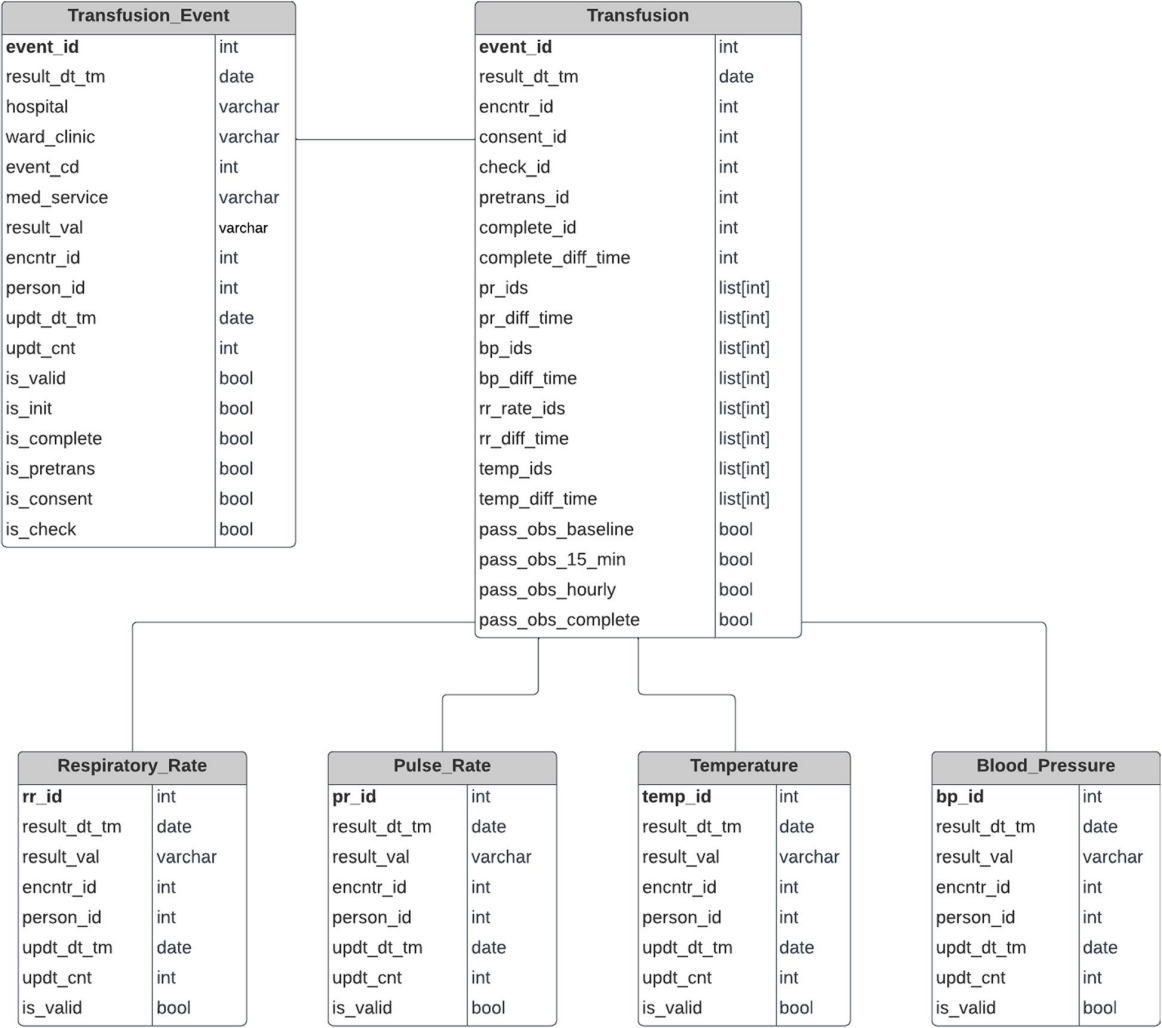
Data Warehouse Schema — Event Granularity. Database model of the highest level of detail. It contains the transformed data at its highest level of granularity

Since the raw data is broken down into individual snapshots of clinical events as shown in Fig. 4, firstly a separation of records is done according to their type. For example, consent records are separated from completion records and pulse rate observations. The second step is to reconstruct each of the transfusions recorded from the previous transformed data. Thus, the designed model displayed in Fig. 5 can hold each of the record categories separately into its own detailed level table. To accomplish this, a Python program was developed to transform and insert the transformed data into its corresponding table (e.g., blood pressure records into blood_pressure table). The partition criteria is based on the value of the “RESULT_NAME” column as seen in Fig. 4. Depending on its value, it will be inserted in its corresponding table. All the tables were created in a PostgreSQL database which is used as the clinical data warehouse.

#### Start and end times of transfusion not linked directly

Given the problem of a transfusion being comprised of several records that need to be recombined, we must reconstruct each instance before performing any analytics. When the Python program preprocesses the new batch of data, it only has the data corresponding to a new period of time. For example, if data is extracted once every hour, it will only have the data for the past hour. Therefore, it wouldn’t be possible to process a complete transfusion since a transfusion could last for hours and the batch of data would miss vital sign observations for the next hours and the record that finishes a transfusion. These records would appear in the next-hour batch of data. Consequently, we can leverage the database engine, which can perform different operations on the data that is being inserted plus the past data that was already inserted. Thus, if a “Complete” record is inserted in the new batch, but the “Initiate” record was inserted in the previous batch, an update operation could match the previously non-closed transfusion with its corresponding “Complete” record. Similarly, the remaining vital sign observations that appear in the latest batch would be allocated to their corresponding transfusion. To process the reconstruction of a blood transfusion, a series of SQL procedures and triggers were defined in the database that will trigger when a “Complete” record is inserted matching with an “Initiate” record.

#### Transfusion event records become invalid

Figure 4 displays a column “UPDT_CNT” that indicates a version of the record. The higher the value, the more recent it is. Therefore, during the preprocessing of the data extract, only latest record will be kept and the previous ones will be filtered out. This processing is done in memory before inserting into the database. However, given that the CDW contains previously transformed data from previous extracts, the new batch might contain a more recent record. Thus, records from the CDW need to be invalidated. To achieve this, a procedure in the database was created to replace the old record with the new record that is being inserted when the unique key constraint is violated.

### Data warehouse

As discussed in the data transformation section, a blood transfusion is reconstructed from the ensemble of individual records. Each record from the extracted dataset contains transfusion events such as consent, complete and vital signs observations. Each of these belong solely to one of the entities (tables) shown in Fig. 5, either “Transfusion_Event” or one of the Vital Sign tables. Only having these tables would not allow descriptive queries such as the following to be easily answered:

Query 1: How many transfusions have missing consents in December 2021?

Query 2: Which hospital branches have the lowest vital sign observations before transfusion?

Therefore the transfusion table is created and where all the reconstructed blood transfusions are stored. Now, each record in this table will contain all the associated information such as the vital sign observations. It also contains some preprocessed metric values like whether a transfusion passes the metric of documented observations for all distinct time periods. Importantly with this schema, it is possible to drill down to see more detailed information given that all associated unique identifiers are stored for each transfusion record.

**Fig. 6:**
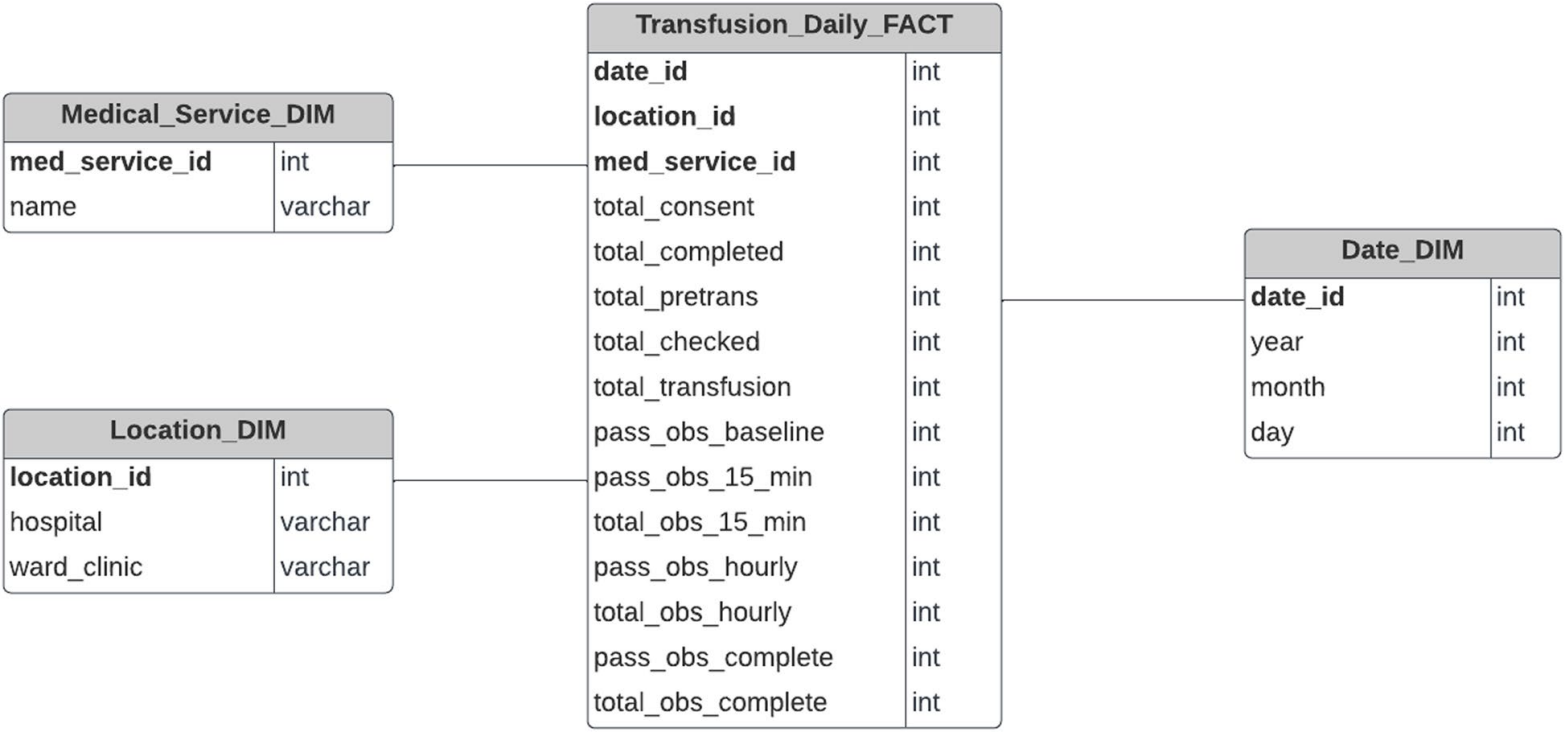
Data Warehouse Schema — Daily Granularity. Database model with aggregated values at a daily level. Designed for the fastest query efficiency for the dashboard

A visualisation tool like a dashboard does not need to show every detail of a transfusion unless it is specifically requested by a user, for example, if a nurse wants to display the information of the blood transfusions from a particular day. A dashboard generally displays aggregated data grouped and/or filtered by the dimensions in its schema. So, it is not necessary that the dashboard client requests all the detailed data to the database. Instead, only precomputed data can be requested and for this, another schema can be built on top of the schema in Fig. 6.

This less-detail schema is represented in Fig. 6. The Transfusion_Daily_FACT table contains all the precomputed values required to calculate the NSQHS Standard metrics in blood management documentation. Furthermore, this schema structure can easily answer the two previous questions (see below) and many others, making it more efficient for analytical queries.

Query 1: How many transfusions have missing transfusion consents in December 2021?
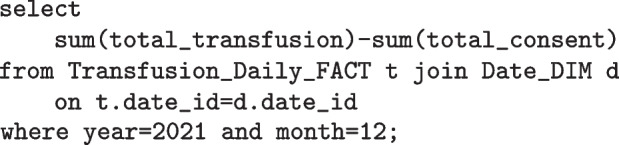


Query 2: Which medical services have the lowest vital sign observations before transfusion on 2021?
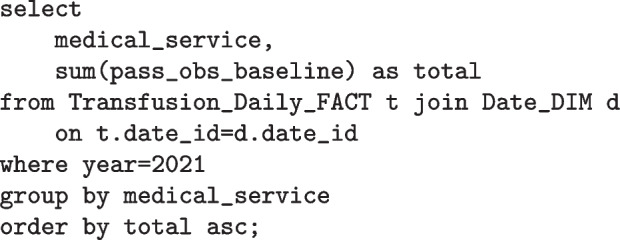


### Data visualisation

The goal of this work is not to come up with SQL queries for several analytical queries because clinicians are unaware of such language and they are mainly interested in the insight that the underlying data can provide. The main purpose is to represent the model in a visual form and also to have the possibility for the clinician to interact with the visual interface and modify the queries with just a simple selection of options in the interface. Nowadays, multiple visualisation tools exist that can leverage the data models implemented in the data warehouse that we build. For this work, an actionable dashboard was created to display the blood transfusion data. The data visualisation was done through Microsoft Power BI and the selection of analytical data was conducted in partnership with stakeholders and clinicians. The selected data comprises NSQHS Standards accreditation measures that have been pre-processed and stored in the data warehouse, such as the consent completion status, EMR documentation completion, blood product compatibility report, blood observations (including the baseline, 15-minute, hourly, and complete observations), and the blood orders.

### Data validation

Data validation was required at all 3 stages of data handling; data extraction, data preprocessing and data visualisation.

Samples of data from data extract reports were cross-checked against the EMR to ensure data points extracted correlated with recorded medical information. Any discrepancies provided a prompt for review and amendment of the CCL queries underpinning the data extract.

Validation of data processing was undertaken by the selection of a small random sample (*n* =15) of unique encounters, and one researcher applying data processing rules manually. These results were then compared to the processed data for concordance. Any discrepancies were discussed with at least two researchers to identify the reason for discordance.

Data visualisation required multiple approaches for data validation. Firstly, a sample (*n* =30) of unique encounters already subject to existing organisational auditing (manual process) was compared to dashboard output for concordance. Any discrepancies were reviewed by at least two researchers. Secondly, researchers together with subject matter experts (Blood nurse specialists at the organisation) reviewed the dashboard output with existing organisational scorecards. Any areas of discordance with expected performance were discussed and a sampling of encounters for cross-checking with the EMR medical records was used, particularly for areas of large discordance.

**Fig. 7:**
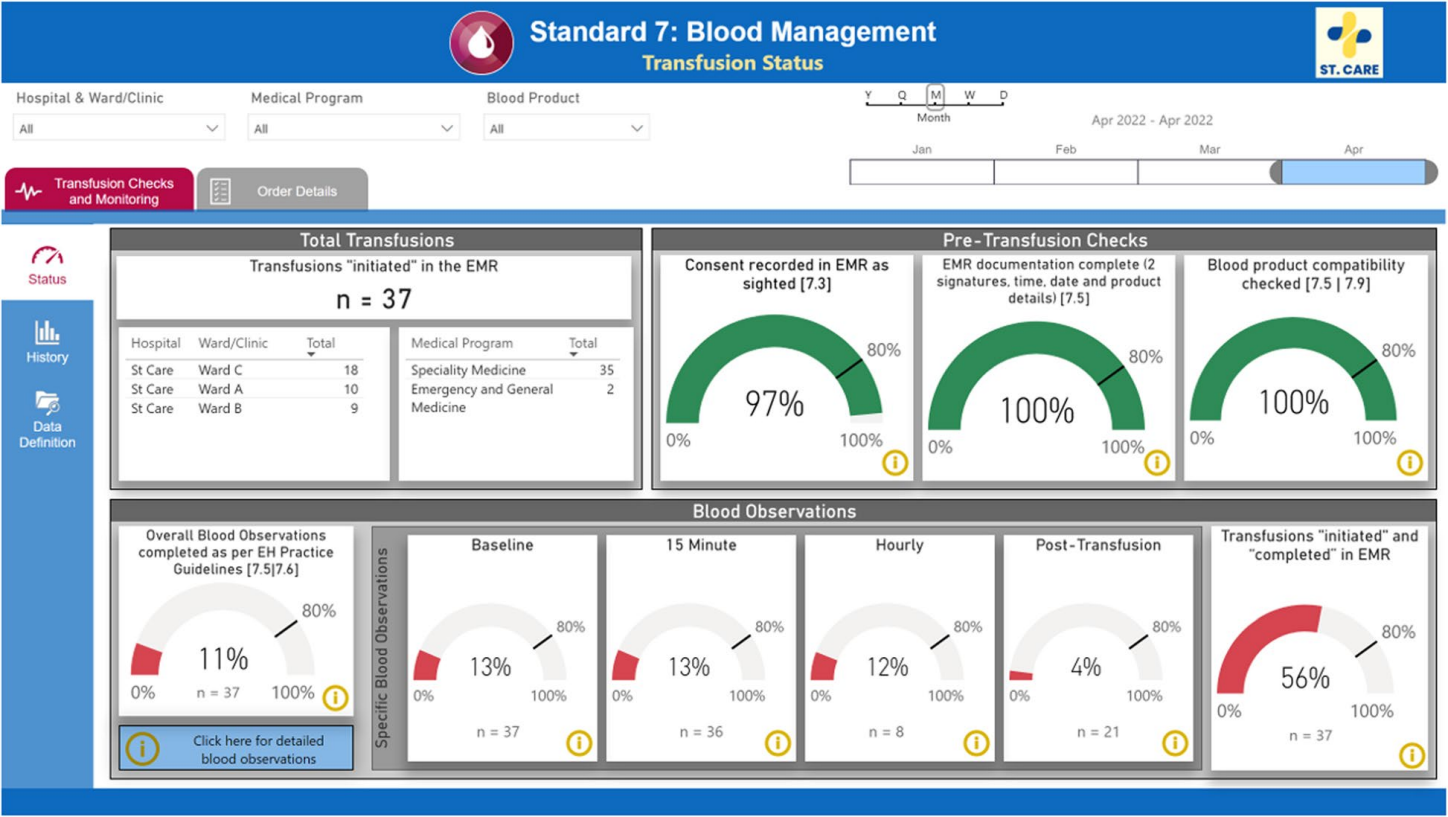
Status View. Displays the current calendar month’s overall performance status for a range of quality measures for blood transfusions

## Results

### Data ingestion

The ETL program that transforms the extracted data from the EMR and loads it into the clinical data warehouse is flexible enough to perform this task at any extraction frequency. From lower frequencies like once per month to higher frequencies such as daily or hourly, the implemented program will execute the logic to maintain the clinical data warehouse up to date.

### Data aggregation

An additional level of detail is implemented and maintained to increase the performance of the analytical queries that the dashboard requires. Initially, there is no need to download the entire historic data from the data warehouse at its most-granular level of detail (individual actions/records), since it will only show aggregated data by day. Therefore, the aggregated level schema shown in Fig. 6, aggregates the data from the most-granular level schema in Fig. 5. This greatly reduces the amount of data that needs to be downloaded initially to the dashboard as well as reducing the amount of time that would otherwise be required to compute the aggregated values. This implementation is flexible and can increase or reduce the number of levels depending on what is commonly shown. For example, if the dashboard needs at first to show data aggregated per month, an additional schema can be implemented with a monthly granularity level. In other words, the schema from the data warehouse can be adjusted to optimise the dashboard requests based on a particular granularity level.

### Dashboard

**Fig. 8:**
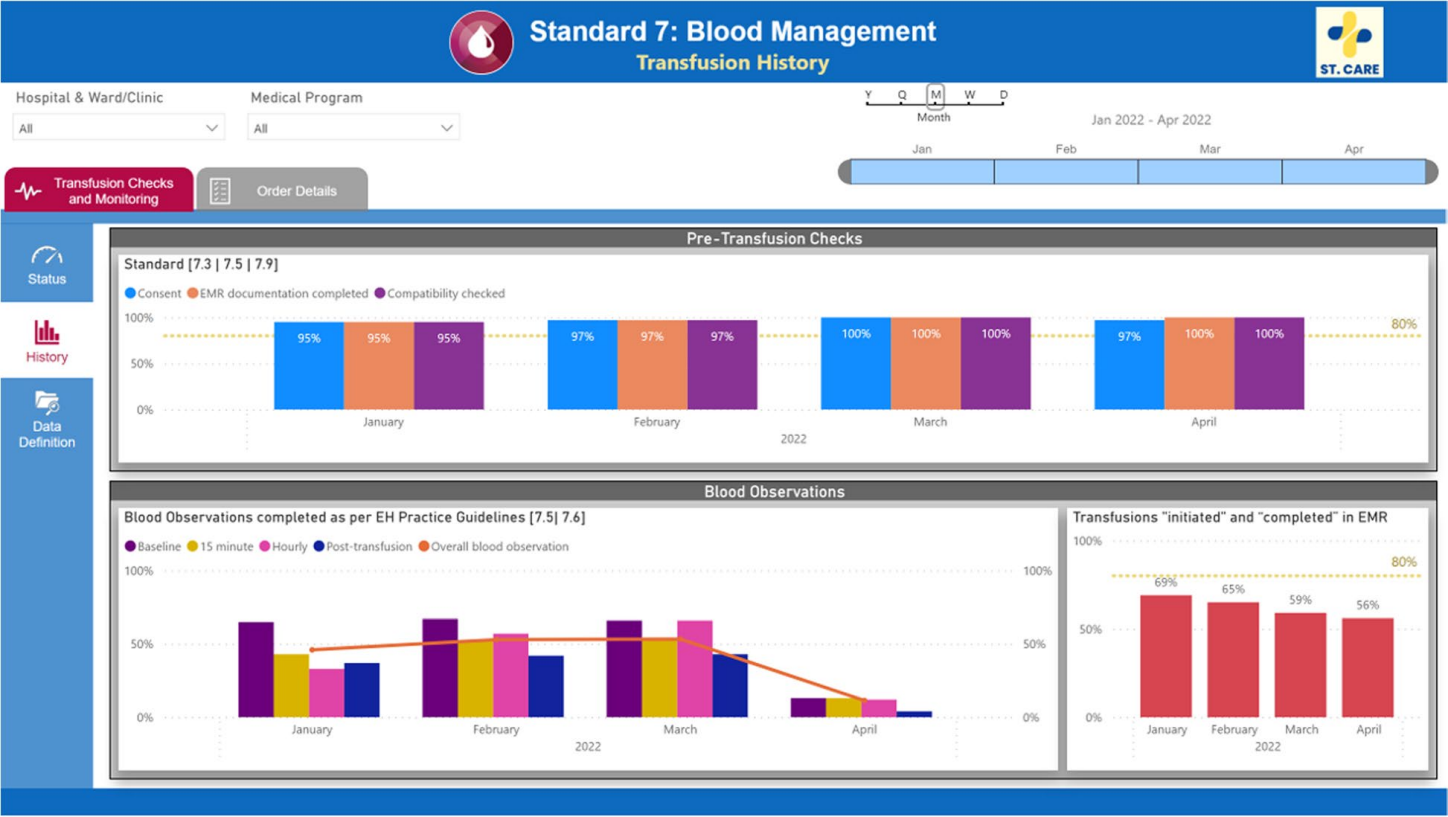
History View. Displays the historical performance for the same quality measures as Fig. 7

The dashboard consumes the processed data from the data warehouse. Once the data is loaded into the business intelligence tool, the selected measures were re-computed and aggregated for analysis purposes. In this case, the dashboard consists of two main information tabs:

- Status (Fig. 7): displays the current calendar month’s status of the selected measures. The measures are shown as gauges and coloured depending on whether targets are being met. For the hospital we worked with, the target for these measures are 80% completion, but these values might differ for other hospitals or other metrics.
- History (Fig. 8): displays all past data (mainly shown in month aggregation).

Several filtering features were included in the dashboard. Users are able to filter the status and history based on the hospital, ward, and medical service. Time filters are also available in which the data can be filtered based on year, month, day, and hour of the day aggregation.

## Conclusions

EMR data, though rich in information, presents challenges due to its structural complexity, hindering direct comprehension and utilisation by humans. To address this, our work introduces a clinical data warehouse tailored for EMR data related to administration of blood products. This data warehouse forms the foundation for a clinical dashboard tailored to blood management data.

In contrast to previous studies, we tackle the difficulties described in the “Data Extraction” section for the blood transfusions data derived from electronic medical records (EMRs). We recognise the complexities of calculating metrics for blood management standards as described in the “Performance Metrics” section, requiring a tailored data preprocessing and data model to calculate the performance metrics required by the national standards. This enables the data to be effectively displayed through dashboard tools like Tableau or Power BI. The contribution of this work is in demonstrating how data warehousing techniques can be utilised to extract and process EMR data in a way that allows the display of near-real-time healthcare accreditation information for clinicians and clinical risk managers. Similarly, the long-format raw dataset requires pre-transformation to extract individual blood transfusions and their associated events, followed by post-transformation in the data warehouse to fully reconstruct the blood transfusion records.

Our presented approach holds significant implications for healthcare accreditation and clinical decision-making at Eastern Health, our partner hospital. Leveraging visual tools from the dashboard, clinicians can enhance care outcomes by documenting and analysing trends in blood transfusions across clinic wards or hospital branches over different time periods. This is especially significant given that following the implementation of this project in our affiliated hospital, all instances of blood transfusions are automatically being taken into account for metrics, compared with a prior healthcare scorecards where data was prepared manually and complex cases such as the ones we address were just omitted in favour of reporting simple cases.

### Limitations

This project focused only on structured data from the EMR system since it was not currently possible to access data from other systems in the hospital. Even though there is unstructured data in the EMR system (e.g., free text case notes) for the metrics that we worked on, it was not necessary to process unstructured data. Furthermore, the data extraction stage is done only for the Cerner EMR system since we did not have the possibility to work with other EMR systems. To implement this work, an exhaustive evaluation of the alternative EMR system would need to be done to find the entities and columns to construct the data extracts that serve as input for the ETL phase.

### Future work

The ingestion of new data is done depending on the frequency of the extraction from the EMR. Therefore, this process does not achieve real-time analysis since it depends on the frequency that extracts are pulled from the EMR. This means that the data is obtained based on the request of the program rather than receiving the data when new blood transfusion records are generated. Furthermore, there are specific metrics from the NHQHS Standards whose information is not located in the EMR but in other systems. In that case, additional data sources could be added and the data warehouse schema would need to be extended with new entities while maintaining the validity of the schema.

## Data Availability

The datasets used for validation are not publicly available due to containing hospital data. The dummy datasets used to showcase the dashboards in Figs. 7 and 8 (which have the same structure as the hospital data) are available from the corresponding author on reasonable request.
